# Genetic Background Influences the Propagation of Tau Pathology in Transgenic Rodent Models of Tauopathy

**DOI:** 10.3389/fnagi.2019.00343

**Published:** 2019-12-11

**Authors:** Tomas Smolek, Veronika Cubinkova, Veronika Brezovakova, Bernadeta Valachova, Peter Szalay, Norbert Zilka, Santosh Jadhav

**Affiliations:** ^1^Institute of Neuroimmunology, Slovak Academy of Sciences, Bratislava, Slovakia; ^2^Axon Neuroscience R&D Services SE, Bratislava, Slovakia

**Keywords:** tauopathy, neurofibrillary tangles, microglia, genetic background, ramified

## Abstract

Alzheimer’s disease (AD), the most common tauopathy, is an age-dependent, progressive neurodegenerative disease. Epidemiological studies implicate the role of genetic background in the onset and progression of AD. Despite mutations in familial AD, several risk factors have been implicated in sporadic AD, of which the onset is unknown. In AD, there is a sequential and hierarchical spread of tau pathology to other brain areas. Studies have strived to understand the factors that influence this characteristic spread. Using transgenic rat models with different genetic backgrounds, we reported that the genetic background may influence the manifestation of neurofibrillary pathology. In this study we investigated whether genetic background has an influence in the spread of tau pathology, using hippocampal inoculations of insoluble tau from AD brains in rodent models of tauopathy with either a spontaneously hypertensive (SHR72) or Wistar-Kyoto (WKY72) genetic background. We observed that insoluble tau from human AD induced AT8-positive neurofibrillary structures in the hippocampus of both lines. However, there was no significant difference in the amount of neurofibrillary structures, but the extent of spread was prominent in the W72 line. On the other hand, we observed significantly higher levels of AT8-positive structures in the parietal and frontal cortical areas in W72 when compared to SHR72. Interestingly, we also observed that the microglia in these brain areas in W72 were predominantly phagocytic in morphology (62.4% in parietal and 47.3% in frontal), while in SHR72 the microglia were either reactive or ramified (67.2% in parietal and 84.7% in frontal). The microglia in the hippocampus and occipital cortex in both lines were reactive or ramified structures. Factors such as gender or age are not responsible for the differences observed in these animals. Put together, our results, for the first time, show that the immune response modulating genetic variability is one of the factors that influences the propagation of tau neurofibrillary pathology.

## Introduction

Alzheimer’s disease (AD), a primary tauopathy, is the most prevalent neurodegenerative disorder characterized by neurodegeneration and dementia. The risk factors for AD include lower mental and physical activity during old age, trauma to the head, cardiovascular diseases, diabetes, smoking, and obesity. Although the role of genetic factors, such as mutation, in early-onset familial AD has been extensively characterized (Campion et al., 1999; Bekris et al., 2010), the exact modality of the onset and progression of the disease pathogenesis in sporadic forms of AD is unknown.

Besides factors such as environmental exposure or psychological factors, epidemiological studies also implicate the role of genetic susceptibility in the onset and progression of sporadic AD (Miech et al., 2002; Cacabelos et al., 2005; Stozicka et al., 2007; Qiu et al., 2009). Genome-wide association studies and their meta-analyses implicate more than 20 genetic loci, mainly involved in the immune system (directly or indirectly related to microglia), lipid metabolism, and endocytosis, as potential risk factors in sporadic late-onset Alzheimer’s disease (Ridge et al., 2016; Jansen et al., 2019; Kunkle et al., 2019). However, clinic-pathological heterogeneity between AD cases complicate the understanding of the role these genetic components play in the manifestation of the disease (Moreno-Grau et al., 2019). It is suggested that genetic susceptibility may influence the effect of environmental and psychological factors on cognitive phenotypes during aging (Wang et al., 2019). Several studies have investigated the influence of genetic susceptibility and environment modifiers on transgenic models of Alzheimer’s disease (Ryman and Lamb, 2006; Stozicka et al., 2010; Zilka et al., 2012b; Pardossi-Piquard et al., 2016). Moreover, we have also demonstrated the influence of genetic background on the manifestation of neurofibrillary pathology and associated neuroinflammation (Stozicka et al., 2010; Zilka et al., 2012b). Despite retaining similar levels of transgene expression, the W72 line showed higher levels of activated microglia than the SHR72 line (Stozicka et al., 2010), suggesting that there exists a strain-dependent difference in response to neurofibrillary pathology.

One of the characteristic features of AD and a few other tauopathies is the propagation of tau pathology in a hierarchical manner to anatomically connected regions (Braak and Braak, 1991; Delacourte et al., 1999). Although studies have, in recent years, strived to recapitulate and understand the modality of the characteristic spread, it is still unknown if genetic background also influences the seeding propensity and propagation of tau pathology. Therefore in this study, we investigated the potency of insoluble tau from human AD to induce tau pathology in well-characterized transgenic rat models of tauopathy expressing human truncated tau aa151–391 in two different genetic backgrounds (Zilka et al., 2006; Koson et al., 2008). Our results show that, regardless of the genetic background, the misfolded tau seeds from AD were able to induce tau pathology; however, genetic background had an influence on the spread of tau pathology in the rodents. To the best of our knowledge, the current study is the first to investigate the role of genetic background in the seeding and propagation of tau pathology.

## Materials and Methods

### Human Brain

Alzheimer’s disease brains (Braak stage 5) were procured from the brain collection of the University of Geneva, Geneva, Switzerland, in compliance with the material transfer agreement. The parietal cortex was used for the isolation of sarkosyl insoluble tau fraction.

### Animals

The study was performed on 3-month-old transgenic female rats expressing human truncated tau aa151–391 with four repeat domains in a spontaneously hypertensive (line SHR72) or Wistar-Kyoto (line W72) genetic background. The SHR72 line was back-crossed to the Wistar-Kyoto genetic background to generate the W72 line, and the normotensive and hypertensive properties of the W72 and SHR72 lines, respectively, have been characterized (Stozicka et al., 2010). The neurofibrillary pathology is localized mainly in the brainstem and spinal cord of these transgenic rats (Zilka et al., 2006; Koson et al., 2008). All animals were housed under standard laboratory conditions with free access to water and food and were kept under diurnal lighting conditions. A total of 12 transgenic animals (*n* = 6/group) were used in the study. Age-matched non-transgenic Wistar-Kyoto (WKY) and spontaneous hypertensive rats (SHR), transgenic W72, and SHR72 lines were used as controls for the morphological examination of Iba-1 positive microglia (*n* = 3/group).

#### Ethics Statement

All experiments were performed in accordance with the Slovak and European Community Guidelines, with the approval of the Institute’s Ethical Committee, and the study was approved by the State Veterinary and Food Administration of the Slovak Republic.

### Isolation of Sarkosyl-Insoluble Tau and Western Blotting

Sarkosyl-insoluble tau was extracted as previously described (Jadhav et al., 2015). Briefly, tissues were homogenized in a buffer containing 20 mM *Tris*, 0.8 M NaCl, 1 mM EGTA, 1 mM EDTA, and 10% sucrose that was supplemented with protease inhibitors (Complete, EDTA free, Roche Diagnostics, United States) and phosphatase inhibitors (1 mM sodium orthovanadate and 20 mM sodium fluoride). After centrifugation at 20,000 × *g* for 20 min, the supernatant (S1) was collected and a small fraction was saved as the total protein extract; 40% w/v of N-lauroylsarcosine (sarkosyl) in water was added to a final concentration of 1% and mixed by stirring it for 1 h at room temperature. The sample was then centrifuged at 100,000 × *g* for 1 h at 25°C using Beckmann TLA-100 (Beckmann instrument Inc., CA, United States). Pellets (P2) were washed and re-suspended in PBS to 1/50 volume of the S1 fraction and sonicated briefly; 10 μg w/v of the P2 fraction corresponding to the S1 fraction was used for the SDS-PAGE analysis.

Western blotting was performed as previously described (Jadhav et al., 2015). Briefly, known amounts of recombinant human tau 2N4R (tau 40) and insoluble tau extract were resolved using 12% SDS-PAGE gels and transferred to nitrocellulose membranes. After blocking, the membranes were incubated using a pan-tau antibody DC25 (1:1 with 5% fat free milk; Axon Neuroscience, Bratislava, Slovakia). Following washing, membranes were incubated with anti-mouse secondary antibody (Dako, Glostrup, Denmark). Blots were developed using a SuperSignal West Pico chemiluminescent Substrate (Thermo Scientific, IL, United States) on an Image Reader LAS-3000 (FUJI Photo Film Co., Ltd., Tokyo, Japan). The semi-quantitative estimation of sarkosyl-insoluble tau was performed as previously described (Smolek et al., 2019). The intensities of the samples and tau 40 were quantified by densitometry using an AIDA Biopackage (Advanced Image Data Analyzer software; Raytest, Germany). The concentration of insoluble tau protein was estimated using a standard curve with reference intensities of known concentrations of recombinant tau 2N4R (Tau 40).

### Stereotaxic Surgery

Rats were anesthetized through intraperitoneal injection of a cocktail containing Zoletil (30 mg/kg) and Xylariem (10 mg/kg). Animals were fixed to a stereotaxic apparatus (Kopf Instruments, CA, United States) and an UltraMicroPump III Micro-syringe injector and Micro4 Controller (World Precision Instruments, FL, United States) were used for applying substances. Animals received 900 ng (concentration 300 ng/ul) of sarkosyl-insoluble tau at a rate of 1.25 μl/minute, and the needle was kept in position for 5 min before slow withdrawal to prevent leakage of the liquid infused. Stereotaxic coordinates for the injection were W72: A/P: −3.6 mm, L: ± 2.0 mm, D/V: 3.1 mm from bregma and SHR72: A/P: −3.6 mm, L: ± 2.0 mm, D/V: 3.3 from bragma (Paxinos and Watson, 1997).

### Immunohistochemistry

Rats were deeply anesthetized before being sacrificed and perfused transcardially with 1× phosphate-buffered saline. Brains were removed and fixed in 4% PFA overnight, followed by permeabilization in sucrose solutions (15, 25, and 30% for 24 h each), freezing in 2-methyl butane, and stored at a temperature of −80°C. Frozen brains were cut serially into sagittal 40 μm-thick sections using a cryomicrotome (Leica CM1850, Leica Biosystems). The sections were blocked with Aptum Section block (Aptum, United Kingdom), and this was followed by incubation with primary antibodies AT8 (epitope anti-tau pS202/pT205, 1:1000; Thermo Scientific, IL, United States), anti-phospho tau pT212 (1:1000; Invitrogen, CA, United States), or Iba1 (1:500; Wako, Japan) overnight at 4°C. Sections were stained with biotin-conjugated secondary antibodies and developed using a Vectastain ABC Kit (Vector Laboratories, CA, United States). After mounting, sections were evaluated using an Olympus BX51 microscope (Olympus microscope solutions). For immunohistological staining, every eighth sagittal section was used; 10 sections were used for quantification and the total distance in range (lateral from the medial line 0.40–3.9 mm). Quantification was performed manually, with every tangle or Iba 1 positive macrophages in the respective regions of interest being counted by a blind observer. Iba 1 positive microglia with two or less processes were considered as phagocytic in morphology.

### Statistical Analysis

Data were analyzed using Prism (Graph Pad Software version 6, CA, United States). An unpaired *t*-test (Mann–Whitney) was used for comparison of histological analysis between two groups. Data are presented as mean ± standard deviation. Differences were considered to be statistically significant if *p* < 0.05. ^∗^*p* < 0.05, ^∗∗^*p* < 0.01, and ^∗∗∗^*p* < 0.001 were used to denote statistical significance.

## Results

### Insoluble Tau From Alzheimer’s Disease Brains Induces Neurofibrillary Pathology in Transgenic Rat Model in a Wistar-Kyoto Background

**FIGURE 1:**
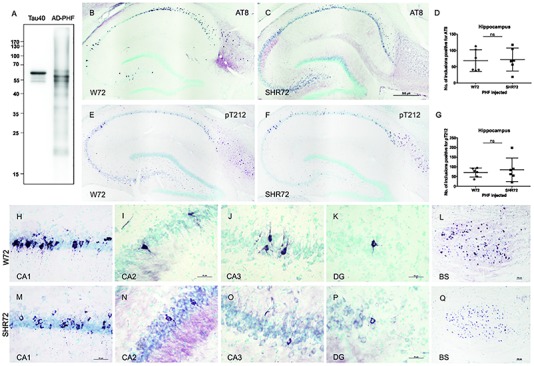
Histological analysis for neurofibrillary pathology in the hippocampus of SHR72 and W72 rats after inoculation of insoluble tau. **(A)** Immuno-blot using pan-tau antibody DC25 showing insoluble tau isolated from human AD brain (AD-PHF). Recombinant human 2N4R (Tau 40) was used as positive control. Histological images showing AT8-positive structures in the hippocampus of W72 **(B, H–L)** and SHR72 transgenic line **(C, M–Q)**. **(E, F)** The presence of neurofibrillary pathology was confirmed using a pT212 phospho-tau specific antibody in the hippocampus of the transgenic lines. A statistical analysis did not reveal any difference in the levels of AT8-positive **(D)** or pT212 positive **(G)** structures in the hippocampus of the two transgenic lines (unpaired *t*-test: AT8 *p* = 0.818; pT212 *p* = 0.999). However, AT8-positive structures were prominent in the CA3 region of the W72 line **(J)** when compared to the SHR72 line **(O)**. There was not difference in the levels of AT8-positive structures in CA1 **(H,M)**, CA2 **(I,N)**, or dentate gyrus **(K,P)**. The brainstem of the transgenic lines was used as a positive control for the presence of genuine AT8-positive structures **(L,Q)**. Scale bar **B**,**C**,**E**,**F:** 500 μm and **H–Q**: 50 μm.

We demonstrated earlier that insoluble tau from the human AD brain induces tau pathology in a transgenic rat model of tauopathy that had an SHR background (Smolek et al., 2019). Therefore, we were intrigued to see whether a similar phenomenon could also be observed in a transgenic rat model with a different genetic background. Similar to SHR72 line, the W72 transgenic rat model also does not develop hippocampal tau pathology despite expressing human truncated tau aa151-391. We performed bilateral injections of insoluble tau from AD brains (900 ng, AD-PHF in Figure 1A) in a transgenic rat model of tauopathy expressing human truncated tau aa151–391 with a Wistar-Kyoto and SHR background at the age of 3 months. After 3 months, we performed histological analyses using phospho-tau specific AT8 and pT212 antibodies. We observed the presence of AT8- and pT212-positive neurofibrillary structures in the hippocampus of transgenic Wistar-Kyoto rats (Figures 1B,E).

We then compared the degree of neurofibrillary pathology in the hippocampus of two transgenic lines after inoculation of insoluble tau from AD brains. Using AT8 (Figures 1B,C,H–Q) and pT212 (Figures 1E,F) antibodies, we did not observe any difference in the levels of neurofibrillary pathology in the hippocampus of both transgenic rats (Figures 1B–G; AT8 *p* = 0.818 and pT212 *p* = 0.999). However, there was a mild difference in the pattern of distribution of neurofibrillary pathology (Figures 1H–K,M–P). In W72, the neurofibrillary pathology displayed more rostral distribution, mainly in CA3 (yet insignificant, *p* > 0.05), from the site of injection when compared to SHR72 (Figures 1J,O). The CA2 (Figures 1I,N) and dentate gyrus (Figures 1K,P) showed identical patterns of distribution. The brainstem, the region where the two transgenic lines innately develop neurofibrillary pathology, was used as a positive control to confirm the presence of genuine AT8-positive staining in our tissue sections (Figures 1L,Q).

### Cortical Distribution of Neurofibrillary Structures Was Markedly Different Between the Two Transgenic Lines

**FIGURE 2:**
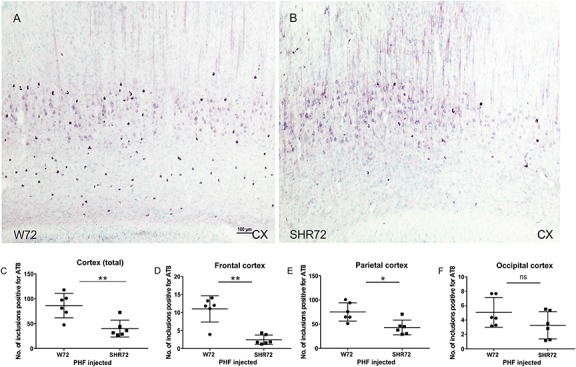
Representative histological images showing AT8-positive neurofibrillary structures in the cortices of the transgenic lines **(A,B)**. **(C,D)** A statistical analysis revealed that the number of AT8-positive structures was higher in the W72 line when compared to SHR72 line (Cortex: *p* = 0.004). **(E)** Higher levels of AT8-positive neurofibrillary tangles were also observed in the frontal cortex (*p* = 0.0043). In the parietal cortex, the levels of AT8-positive structures were higher than the SHR72 line (*p* = 0.0152). **(F)** The occipital cortex showed moderately higher levels of AT8-positive structures; this was, however, insignificant (*p* = 0.2251). ^∗^*p* < 0.05 and ^∗∗^*p* < 0.01 is used to denote statistical significance. An unpaired *t*-test was used for statistical analysis. Scale bar: 100 μm.

We proceeded to observe if the inoculation of insoluble tau induced neurofibrillary pathology in adjacent cortical areas. We observed neurofibrillary pathology in cortices of both transgenic lines; however, the extent of neurofibrillary pathology was different between the two lines. We observed that the number of AT8-positive structures were higher in the cortex of W72 transgenic rats (Figures 2A,C; *p* = 0.004), than in the SHR72 line (Figures 2B,C). The frontal cortex also showed higher levels of neurofibrillary structures in W72 when compared to the SHR72 line (Figure 2D; *p* = 0.0043). The parietal cortex also demonstrated higher levels of AT8-positive structures then the SHR72 (*p* = 0.0152) (Figure 2E). The occipital cortex displayed a tendency to develop higher neurofibrillary pathology in the W72 line but was not statistically significant (Figure 2F; *p* = 0.2251).

### Microglia Displayed Morphological Differences in Regions With Neurofibrillary Pathology Between the Two Transgenic Lines

**FIGURE 3:**
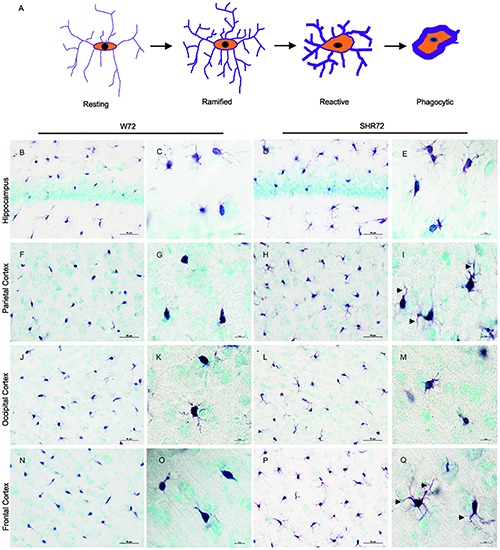
Histological images showing Iba1 positive microglia in the hippocampus and cortical regions. **(A)** Schematic illustration showing changes in microglial morphology in response to stimuli. Upon activation, microglia become hyper-ramified, developing numerous processes, and subsequently become reactive and phagocytic. Iba1-positive structures in the hippocampus of the W72 line **(B,C)** and SHR72 **(D,E)** revealed microglia with both reactive and ramified morphology in both lines (W72: 17.8% and SHR72: 16.6%). However, as evidenced by the size and form of the soma, the microglia showed phagocytic morphology in the parietal cortex **(F,G)** and frontal cortex **(N,O)** of the W72 line (62.4 and 47.3%, respectively). The microglia from the SHR72 were predominantly displaying reactive or ramified morphology, with numerous processes (arrowheads) in the cortex **(H,I)**–the frontal cortex in particular (**P,Q**; 84.7%). The microglia in occipital cortex from both lines were ramified or phagocytic in morphology (**J–M**; W72: 53.6% and SHR72: 52.1% of phagocytic microglia). Scale bar **B**,**D**,**F**,**H**,**J**,**L**,**N**,**P**; 50 μm, rest 10 μm.

We previously reported that genetic background-mediated immunomodulation, dependent on microglia, plays a role in the development of neurofibrillary pathology in these transgenic lines (Stozicka et al., 2010; Zilka et al., 2012b). We therefore used anti-Iba 1 antibody to investigate the microglial status between the two transgenic lines after the induction of tau pathology. Upon response to stimuli, microglia undergoes morphological changes (Figure 3A; Streit et al., 1999). Initially, there was an increase in the ramification of microglial processes (ramified state), followed by hypertrophy (reactive phase). In the end, the microglia became phagocytic, characterized by de-ramification, with few or no processes (Figure 3A).

In the hippocampus of both transgenic lines, the anti-Iba1 antibody revealed microglia with ramified or phagocytic morphology (Figures 3B–E). However, we did not observe any differences in microglial morphology in the hippocampus between the two lines (percentage of phagocytic microglia: W72: 17.8% vs. SHR72: 16.6%). On the other hand, the anti-Iba1 antibody revealed a marked difference in the microglial morphology of the cortex between the two transgenic lines. In the parietal cortex (Figures 3F,G) and frontal cortex (Figures 3N,O) of the W72 line, regions that develop significantly higher levels of neurofibrillary pathology, the majority of microglia either displayed phagocytic morphology with few degenerating processes or were completely devoid of them. In comparison, the microglia in those regions in the SHR72 line prominently displayed ramified to reactive morphology with numerous digitating processes (Figures 3H,I,P,Q). The levels of phagocytic microglia in the parietal cortex in the W72 and SHR72 lines were 62.4 and 32.7%, respectively. In the frontal cortex, the levels were 47.3% in W72 and 15.2% in SHR72 (W72 vs. SHR72). Interestingly, we did not observe any differences in the microglial morphology in the occipital cortex, which displayed either reactive or ramified microglia in both lines (Figures 3J–M) (phagocytic microglia: W72: 53.6% and SHR72: 52.11%). We did not observe any difference in the microglial morphology of non-transgenic Wistar-Kyoto, SHR, and transgenic non-injected W72 and SHR72 in any of the examined regions (Supplementary Figures S1, S2). This suggested that there was a genuine difference in morphology in Iba 1 positive microglia after inoculation of exogenous tau seeds between the two transgenic lines in the study.

## Discussion

In this study, using two transgenic rat models of tauopathy, we demonstrated the influence of genetic background on the induction and propagation of tau pathology. In our earlier study (Stozicka et al., 2010), we observed cardinal differences in Iba1-positive microglia between the two transgenic lines. Therefore, we only used the transgenic lines in the study. In order to avoid bias, we utilized our well-established transgenic rodent models (Zilka et al., 2006; Koson et al., 2008; Stozicka et al., 2010) expressing the same human truncated tau aa151–391 under the same type of promoter in the study. The two animal models had the same copy number of transgenes in their genome and had similar expression levels of truncated tau. The transgenic rat models of tauopathy replicated crucial features of Alzheimer’s disease, such as tau hyperphosphorylation, insoluble tau formation, neurofibrillary pathology, and reduction in life span (Zilka et al., 2006; Koson et al., 2008). The animal models did not develop neurofibrillary pathology in the hippocampus. In order to induce tau pathology, we injected insoluble tau from a human AD brain into the hippocampus. We recently demonstrated that inoculation of insoluble tau from an AD brain induced neurofibrillary pathology in the hippocampus of SHR72 transgenic rats (Smolek et al., 2019). Moreover, the neurofibrillary pathology spread to other regions from the site of injection and induced misfolding of endogenous rat tau, culminating in inclusions containing both exogenous and endogenous tau species (Smolek et al., 2019). We therefore used a similar approach to investigate the influence of genetic background on the propagation of tau pathology.

We first showed that the inoculation of tau seeds from a human AD brain induced neurofibrillary pathology in the hippocampus of the W72 line, similar to SHR72. However, we observed that the lines demonstrated significant differences in the propagation of tau neurofibrillary pathology. We observed that the W72 line developed more pathology in the rostral cortical areas from the hippocampus, even in the frontal cortex, when compared to SHR72 line. We previously reported that W72, the animal model with higher levels of activated microglia, were more susceptible to neurofibrillary degeneration (Stozicka et al., 2010). Our results further extended these observations and showed that the W72 line is also more susceptible to propagation of tau pathology than the SHR72 line.

Studies have observed that the presence of neurofibrillary inclusions corresponded to the distribution of active microglia in AD (Sheng et al., 1997; Sheffield et al., 2000), and activated microglia were frequently present in close proximity to neurofibrillary tangles (Cras et al., 1991; DiPatre and Gelman, 1997; Zilka et al., 2012b). Studies show that region-specific microglial morphology has been observed in models of neuroinflammation (Fernandez-Arjona et al., 2017) and stress (Avignone et al., 2015). Interestingly, we also observed major differences in the morphology of microglia in both transgenic lines in response to induced neurofibrillary pathology. In the W72 line, the cortical areas with higher levels of neurofibrillary pathology than SHR72 showed higher levels of microglia demonstrating phagocytic morphology. On the other hand, most microglia showed ramified or reactive morphology in the SHR72 line. Factors such as age or gender were not responsible for the difference in microglial morphology in our study since the experiments were performed on animals of the same age and gender and for the same period. Therefore, the distinct responses of microglia between the two transgenic lines may be due to their unique genetic background, as we previously described (Stozicka et al., 2010).

Despite the presence of active phagocytic microglia in the W72, we observed significantly higher levels of neurofibrillary pathology in this line, mainly in the cortical areas. We have previously reported that the levels of neurofibrillary tangles correlated well with the levels of activated microglia in transgenic model of tauopathy (Stozicka et al., 2010). Studies have also shown that microglia-mediated inflammatory responses may contribute to the development of tau pathology and thus accelerate the course of disease (Zilka et al., 2012b; Maphis et al., 2015). Furthermore, amelioration of AD pathology using immunomodulatory agents in transgenic models of AD has been shown to inhibit the inflammatory consequences of tau aggregation (Martinez and Peplow, 2019). Numerous studies have also implicated the direct role of microglia in tau propagation (Asai et al., 2015; Maphis et al., 2015). A recent study evidenced the presence of competent microglia capable of the uptake and release of tau in an AD brain and transgenic model of tauopathy (Hopp et al., 2018); however, the microglia were only partially able to neutralize the seed-competent tau. Since we observed higher levels of phagocytic microglia in the W72 line, in areas accompanied by higher levels of tau pathology, we speculate that strain-dependent role of microglia may be responsible for the manifestation of higher levels of neurofibrillary pathology in the cortices of W72 line.

In the end, the rat lines were developed based on an SHR or Wistar-Kyoto background, and the two lines are genetically different (H’Doubler et al., 1991). Interestingly, chronic inflammation in SHR probably causes an altered reaction to acute immune challenges (Bernard et al., 1998; Bauhofer et al., 2003). The SHR rats demonstrated resistance to LPS stimuli and had better survival rates than the Wistar-Kyoto counterparts. This attenuated response was independent of hypertension in these rats but was due to an altered inflammatory response (Bernard et al., 1998). In our previous study, we demonstrated that the WKY tg rats developed a more robust immunologic response in comparison to SHR tg rats (Stozicka et al., 2010). Therefore, the overall response to exogenous tau in SHR tg or WKY tg to tau spreading in our study was independent of the hypertension and was dependent on the difference in immunological susceptibility between the two lines. Although hypertension is considered a risk factor, inflammation may have a more prominent role in the progression of pathology in neurodegenerative tauopathies.

Several studies have discussed the interrelationship between the immune response and tau neurofibrillary pathology (Zilka et al., 2012a; Perea et al., 2018; Spanic et al., 2019). Reactive microglia are associated with neurofibrillary tangles, suggesting that the tau can trigger neuroinflammation. However, the exact relationship between tau propagation and the immune response is less known. Interestingly, higher levels of microglia were observed in hippocampus CA1, the entorhinal cortex, and the parasubiculum in humans (Sheffield et al., 2000)—the regions that demonstrate propagation of tau pathology in AD brains during disease progression. Similar distributions of microglia were also observed in the mouse model of tauopathy, where microglial activation preceded tangle formation (Yoshiyama et al., 2007). Based on this evidence, one can speculate that the regional distribution of microglia and its activation may contribute to propagation of tau pathology. Our study demonstrates that, irrespective of the presence of microglia, the genetic background had an influence on the microglial activation status and, subsequently, the propagation of tau pathology.

Several factors are responsible for the propagation of tau pathology *in vivo*, including genetic factors (Ryman and Lamb, 2006; McQuade and Blurton-Jones, 2019), the presence of a proper seeding partner in the host (Levarska et al., 2013), and the stability and inter-individual variability of the tau strains (Smolek et al., 2019). Our results further extended these observations and showed, for the first time, that the immune response modifying the genetic background is one of the factors that also has an influence on the susceptibility to, and propagation of, tau neurofibrillary pathology in AD and other tauopathies. Our study also demonstrated, for first time, the strain-dependent response of microglia to neurofibrillary pathology in a rodent model of tauopathies. The study opens new vistas in our understanding of the pathogenesis of neurodegenerative disorders, such as Alzheimer’s disease and other tauopathies.

## Data Availability Statement

The datasets generated for this study are available on request to the corresponding author.

## Ethics Statement

The animal study was reviewed and approved by Ethical Committee of Institute of Neuroimmunology, Slovak Academy of Sciences and State Veterinary and Food Administration of the Slovak Republic.

## Author Contributions

TS performed the histological analysis and imaging. VC performed the stereological injections. VB and SJ performed the isolation and characterization of insoluble tau. BV was involved in the handling of animals and stereological analysis. PS was involved in the histological analysis. SJ supervised the project. NZ and SJ wrote the manuscript. All authors approved the final manuscript.

## Conflict of Interest

TS, VC, BV, PS, NZ, and SJ are employees of Axon Neuroscience R&D Services SE.

The remaining author declares that the research was conducted in the absence of any commercial or financial relationships that could be construed as a potential conflict of interest.
