# Corrigendum: Genetic Background Influences the Propagation of Tau Pathology in Transgenic Rodent Models of Tauopathy

**DOI:** 10.3389/fnagi.2020.00030

**Published:** 2020-02-18

**Authors:** Tomas Smolek, Veronika Cubinkova, Veronika Brezovakova, Bernadeta Valachova, Peter Szalay, Norbert Zilka, Santosh Jadhav

**Affiliations:** ^1^Institute of Neuroimmunology, Slovak Academy of Sciences, Bratislava, Slovakia; ^2^Axon Neuroscience R&D Services SE, Bratislava, Slovakia

**Keywords:** tauopathy, neurofibrillary tangles, microglia, genetic background, ramified

There is an error in the Funding statement. The correct number for the first **APVV** grant is **APVV-17-0642**. The corrected statement appears below.

## Funding

The work was supported by research grants APVV-17-0642, APVV-18-0515, VEGA 2/0181/17 VEGA 2/0135/18, and VEGA 2/0167/17.

The authors apologize for this error and state that this does not change the scientific conclusions of the article in any way. The original article has been updated.

