# Supplementary figures and images for: Genetic Background Influences the Propagation of Tau Pathology in Transgenic Rodent Models of Tauopathy
Source: Front Aging Neurosci. 2019 Dec 11;11:343. doi: 10.3389/fnagi.2019.00343 (PMC6917578; doi:10.3389/fnagi.2019.00343)

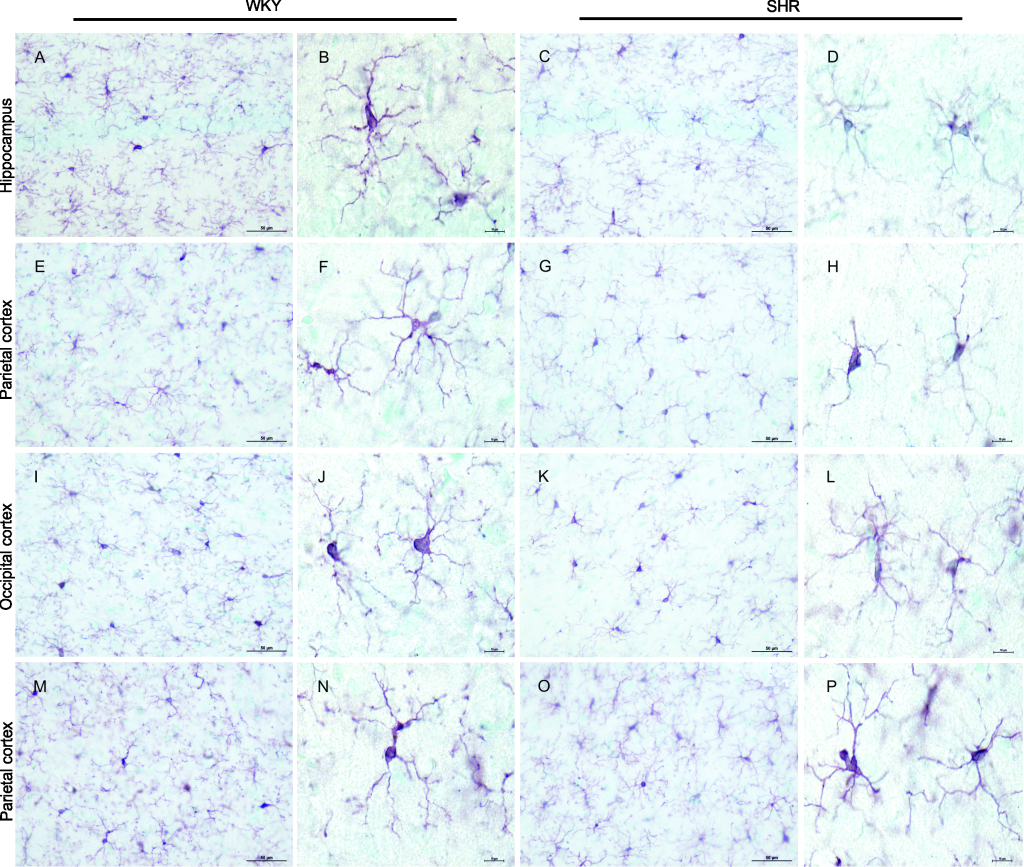

Supplement: Supplementary file 1 [file Image_1.TIF]

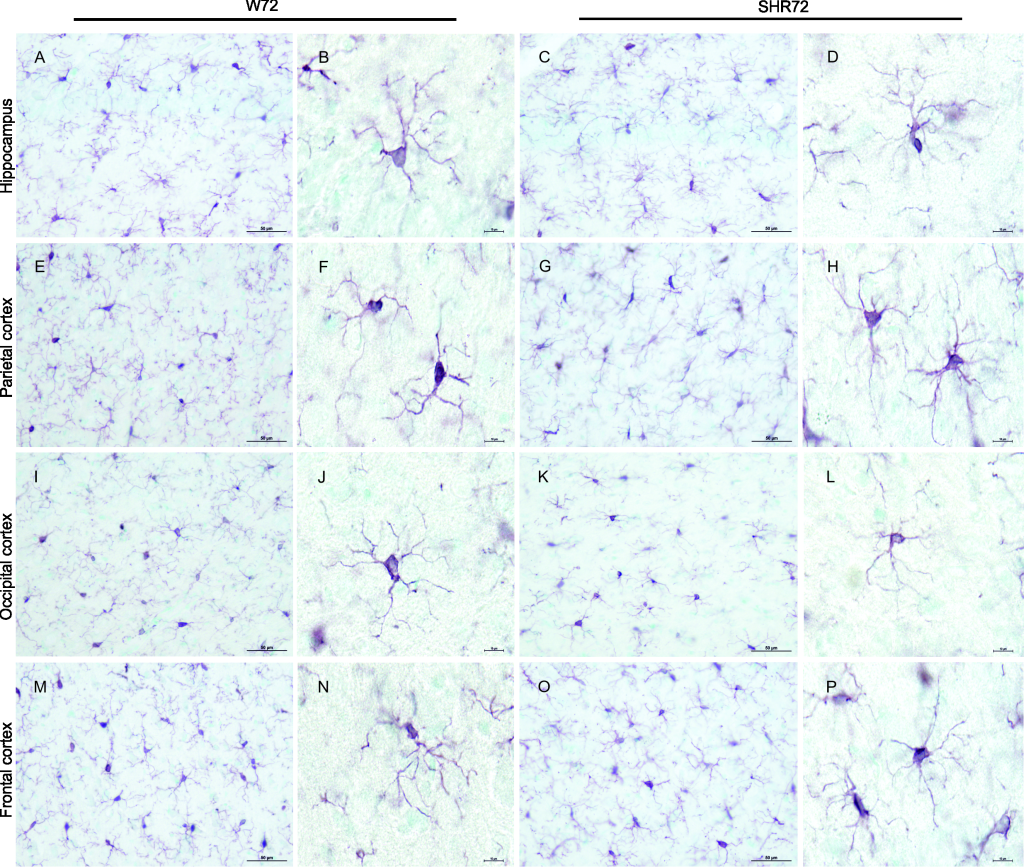

Supplement: Supplementary file 2 [file Image_2.TIF]
