# Supplementary material for: Genetic Background Influences the Propagation of Tau Pathology in Transgenic Rodent Models of Tauopathy
Source: Front Aging Neurosci. 2019 Dec 11;11:343. doi: 10.3389/fnagi.2019.00343 (PMC6917578; doi:10.3389/fnagi.2019.00343)
Supplement: Supplementary file 3 [file Table_1.DOCX]

**Supplementary figure legends**

**Supplementary figure 1.** Iba1 positive microglia in wistar-Kyoto (WKY) and spontaneous hypertensive rats. Histological images showing Iba1 positive microglia in hippocampus of Wistar-Kyoto (A,B) and spontaneous hypertensive rats (SHR) (C,D), parietal cortex (WKY: E,F and SHR: G,H), Occipital cortex (WKY: I,J and SHR: K,L), and frontal cortex (WKY: M,N and SHR: O,P). Scale bar: A,C,E,G,J,K,M,O: 50 µm; rest: 10 µm.

**Supplementary figure 2.** Iba1 positive microglial in uninjected W72 and SHR72 transgenic rats. Histological images showing Iba1 positive microglia in hippocampus of W72 (A,B) and SHR72 (C,D), parietal cortex (W72: E,F and SHR72: G,H), Occipital cortex (W72: I,J and SHR72: K,L), and frontal cortex (W72: M,N and SHR72: O,P). Scale bar: A,C,E,G,J,K,M,O: 50 µm; rest: 10 µm.
